# Follistatin Attenuates Myocardial Fibrosis in Diabetic Cardiomyopathy *via* the TGF-β–Smad3 Pathway

**DOI:** 10.3389/fphar.2021.683335

**Published:** 2021-07-27

**Authors:** Yinhui Wang, Kun Yu, Chengcheng Zhao, Ling Zhou, Jia Cheng, Dao Wen Wang, Chunxia Zhao

**Affiliations:** Hubei Key Laboratory of Genetics and Molecular Mechanisms of Cardiological Disorders, Division of Cardiology, Department of Internal Medicine, Tongji Hospital, Tongji Medical College, Huazhong University of Science and Technology, Wuhan, China

**Keywords:** follistatin, diabetic cardiomyopathy, fibrosis, TGF-β, hypertrophy

## Abstract

Follistatin (FST) is an endogenous protein that irreversibly inhibits TGF-β superfamily members and plays an anti-fibrotic role in other diseases. However, the role of FST in diabetic cardiomyopathy remains unclear. In this study, we investigated the effects of FST on diabetic cardiomyopathy. The expression of FST was downregulated in the hearts of db/db mice. Remarkably, overexpressing FST efficiently protected against cardiac dysfunction. In addition, overexpression of FST promoted cardiac hypertrophy with an unchanged expression of atrial natriuretic peptide (ANP) and the ratio of myosin heavy chain-β/myosin heavy chain-α (MYH7/MYH6). Furthermore, FST reduced cardiac fibrosis and the production of reactive oxygen species (ROS), and enhanced matrix metallopeptidase 9 (MMP9) activities in db/db mouse hearts. We also observed that overexpressing FST decreased the level of transforming growth factor beta (TGF-β) superfamily members and the phosphorylation of Smad3; consistently, *in vitro* experiments also verified the above results. Our findings revealed the cardioprotective role of FST in attenuating diabetic cardiomyopathy through its anti-fibrotic effects through the TGF-β–Smad3 pathway and provided a promising therapeutic strategy for diabetic cardiomyopathy.

## Introduction

The prevalence of patients with diabetes mellitus is rapidly increasing worldwide and is currently estimated to be 11.6% in China (Xu et al., 2013) and 8.3% in America (Bugger and Abel, 2014). Cardiovascular disease is the leading cause of death in the diabetic population. Diabetes cardiomyopathy, as one of the main cardiovascular complications of type 2 diabetes, is strongly associated with a high mortality rate in diabetic patients (Dei Cas et al., 2015).

Diabetic cardiomyopathy is a special cardiomyopathy that occurs in diabetic patients without coronary artery disease, hypertension, or other heart diseases. It is characterized by early diastolic dysfunction, accompanied by cardiac hypertrophy, myocardial fibrosis, and cardiomyocyte apoptosis (Fein, 1990; Huynh et al., 2014). Multiple mechanisms involved in the progression of diabetic heart have been reported, including mitochondrial dysfunction, oxidative stress, advanced glycation end products (AGEs), impairment of mitochondrial Ca2+ handling, inflammation, activated renin-angiotensin-aldosterone system(RAAS), autonomic neuropathy, endoplasmic reticulum stress, cardiomyocyte death, and microvascular dysfunction (Sung et al., 2015; Faria and Persaud, 2017; Jia et al., 2018). Previous studies have suggested that increased fibrosis is observed in both human samples and animal models suffering from diabetic cardiomyopathy and plays a critical role in the progression of pathological structure and functional changes in diabetic cardiomyopathy (Shimizu et al., 1993; Mizushige et al., 2000; Westermann et al., 2007; Linthout et al., 2008). Interstitial fibrosis induced by hyperglycemia disturbs the normal homeostasis between the synthesis and degradation of matrix proteins, causing excessive accumulation of the extracellular matrix (ECM) and alteration of the structure and function of matrix proteins, which leads to cardiac stiffness and impairment of cardiac contractile function (Talior-Volodarsky et al., 2012; Joshi et al., 2014).

Similar to cardiac fibrosis, oxidative stress contributes to the development of diabetic cardiomyopathy. Numerous studies have shown that oxidative stress increases significantly in response to hyperglycemia in the vascular tissues of diabetic patients (Natali et al., 2017; Lytrivi et al., 2018). Interestingly, fibrosis and oxidative stress can be regulated by the TGF-β signaling pathway. TGF-β activation promotes myofibroblast differentiation, upregulates the level of extracellular matrix (ECM), and reduces the expression of matrix metalloproteinases (MMPs), which specifically restrain the ECM (Yuan Ma et al., 2017). Another study proved that the loss of Smad3, downstream of the TGF-β signaling pathway, reduces cardiac fibrosis and improves cardiac compliance (Biernacka et al., 2015). In addition, overexpression of TGF-β triggers cell oxidative stress through upregulation of Nox4 by inhibiting manganese-dependent superoxide dismutase (MnSOD) (Michaeloudes et al., 2011). Therefore, anti-fibrotic activity and antioxidative stress may prevent the development of diabetic cardiomyopathy by inhibiting the excessive activation of TGF-β.

Follistatin (also known as FST), an endogenous blocker of the TGF-β signaling pathway, is broadly expressed in tissues. FST plays a blocking role mainly by binding activins and other TGF-β superfamily proteins, including growth differentiation factor 8 (GDF8), growth differentiation factor 9 (GDF9), and a number of bone morphogenic proteins (BMPs 2, 5, 7, and 8) (Lin et al., 2003). Some evidence has confirmed the anti-fibrotic effect of FST in inhibiting activin A, but most evidence has mainly focused on fibrosis of the liver, kidney, lung, and pancreas, and wound healing (Ohnishi et al., 2003; Aoki et al., 2005; Patella et al., 2006;Pons et al., 2018). Other studies have shown that FST reduces ROS production by neutralizing activin and might attenuate the progression of diseases such as chronic kidney disease, sarcopenia, and preeclampsia (Zhang et al., 2018; Mehta et al., 2019). However, the effects of FST on myocardial fibrosis and oxidative stress in diabetic cardiomyopathy have been rarely reported.

Therefore, we hypothesized that FST could improve the development of diabetic cardiomyopathy by reducing the production of ROS and fibrosis by inhibiting the TGF-β signaling pathway. Our results suggested that FST could protect cardiac function by reducing oxidative stress and fibrosis in db/db mice.

## Research Design and Method

### Patients

Seventeen diabetic patients with complications of heart failure from Tongji Hospital, Tongji Medical College of Huazhong University of Science and Technology, and thirteen healthy volunteers were enrolled in this study. Serum from these people was collected for this study. In accordance with the Declaration of Helsinki, this study was approved by the Ethics Committee of Tongji Hospital, and informed consent was obtained from all subjects prior to study initiation.

### Overexpression of FST in the Myocardium

Custom-made rAAV (type 9) combined with the cardiac troponin T (cTnT) promoter and full-length cDNA of mouse FST or GFP was purchased from Hanbio Biotechnology Co. Ltd. (Shanghai, China). This recombinant adeno-associated virus was injected into the mice through the tail vein to overexpress FST.

### Animals

All animal studies were performed with the approval of the Animal Research Committee of Tongji Medical College and in adherence with the Guide for the Care and Use of Laboratory Animals published by the NIH. For *in vivo* studies, male db/db mice on a C57BLKS (BKS) background and control C57BLKS (BKS) mice (Model Animal Research Center of Nanjing University, Nanjing, China) were used. All animals were maintained on a 12-h light/12-h dark cycle and fed standard chow *ad libitum*. Mice were randomly divided into four groups (con + AAV9-GFP, con + AAV9-FST, db/db + AAV9-GFP, and db/db + AAV9-FST, *n* = 8 each group). All mice were injected with rAAVs (dose: 100 µL/mouse; titer: 1 × 10^12 v.g/mL) *via* the tail vein at the age of 20 weeks and sacrificed at 28 weeks. Heart tissue samples were snap-frozen in liquid nitrogen or collected for paraffin embedding.

### Glucose Tolerance Tests

When the mice were 28 weeks old, OGTTs were used. The mice were fasted but had free access to water for 16 h before the procedure. At the start of the procedure, the mice were weighed, and basal glucose levels were measured. Glucose levels were measured again after oral glucose administration (2 g/kg) at 15, 30, 60, and 120 min.

### Echocardiography

Transthoracic echocardiography was performed 8 weeks after injection of rAAVs using a 30-MHz high-frequency scan head (VisualSonics Vevo770; VisualSonics, Toronto, ON, Canada) as previously described (Yan et al., 2015). All of the obtained data were averages from at least three cycles per loop.

### Hemodynamic Analysis

The hemodynamics of the mice were assessed as previously described (Dai et al., 2018) when the mice were 28 weeks old. Briefly, the right carotid artery was isolated from the surrounding tissue, and then a 1.0-Fr Millar Mikro-Tip catheter transducer (Millar 1.4F, SPR 835, Millar Instruments, Houston, TX) connected to a pressure transducer (Millar Instruments, Houston, TX) was inserted through the right carotid artery into the LV cavity. Hemodynamic parameters were recorded and analyzed with PowerLab and LabChart software. All of the measurement data were obtained from at least three measurements.

### Palmitate Preparation

Palmitate (PA) solutions were prepared as described previously (Song et al., 2012). Briefly, 100 mM palmitate (Sigma, P9767, Shanghai, China) stocks were prepared in 0.1 M NaOH at 70°C and filtered. A twenty percent (weight/volume) palmitate-free BSA (Sigma, A2153, Shanghai, China) solution was prepared in serum-free DMEM. After the palmitate dissolved, the palmitate solutions were added to serum-free DMEM containing BSA. The 25 mM palmitate/20% BSA solution was prepared by complexing appropriate amounts of palmitate to 20% BSA in a 40°C water bath.

### Cell Culture and Treatment

Human cardiomyocytes (AC16) were purchased from ScienCell (San Diego, CA, Catalog Number: 6200). Cells were carefully cultured according to the product instructions and were treated with palmitate (250 μM) and high glucose (33 mM) for 24 h.

NIH3T3 cells were purchased from ATCC (Manassas, VA). NIH3T3 cells were cultured in DMEM with glucose (5.5 mmol/L normal glucose) supplemented with 10% fetal bovine serum at 37°C in a CO_2_ incubator. After starvation in serum-free medium for 24 h, the NIH3T3 cells were incubated in DMEM containing 5.5 mmol/L glucose, 33 mmol/L glucose (high glucose, HG), HG plus 10 ng/ml recombinant FST protein (human recombinant follistatin, BioVision, #4708), or HG plus 10 ng/ml recombinant FST protein and 20 μM TGF-β1 (MedChemExpress, United States). After incubation at 37°C for 24 h, the cells were collected.

### Western Blotting

Total heart tissue homogenate and cell proteins (20 µg) were separated on a 10% SDS-PAGE gel, transferred onto PVDF membranes, and blocked with 5% nonfat dry milk in TBS-T for 2 h. Then, membranes were incubated overnight at 4°C with primary antibodies, followed by peroxidase-conjugated secondary antibody for 1 h, and finally developed with the ECL system (Beyotime Institute of Biotechnology, Nanjing, China). Antibodies against phospho-AKT (Thr308) (catalog no. 13038), AKT (catalog no. 9272), phospho-Smad3 (Ser423/425) (catalog no. 9520), Smad3 (C67H9) (catalog no. 9523), and COL1A1 (catalog no. 84336) were purchased from Cell Signaling Technology (Shanghai, China). Anti-GAPDH (catalog no. AC002) and anti-CTGF (catalog no. A11067) antibodies were purchased from ABclonal Technology (Wuhan, China). Anti-FST (catalog no. 60060-1-Ig) and anti-COL3 (catalog no. 22734-1-AP) antibodies were purchased from Proteintech Group (Wuhan, China). Western blotting results were quantified by densitometry and processed with ImageJ software (National Institutes of Health software).

### RNA Extraction and Real-Time PCR

Total RNA from frozen tissues or cells was extracted using TRIzol (Invitrogen, Carlsbad, CA, United States) according to the manufacturer’s protocol. Then, total RNA was reverse-transcribed with MultiScribe Reverse Transcriptase (Thermo Scientific, Carlsbad, CA). The primers for gene PCR were synthesized by TsingKe (Wuhan, China), and their sequences are listed in Supplemental Table S2. The amplification and detection of specific gene products were performed on a 7900HT Fast Real-Time PCR system (Applied Biosystems, Foster City, CA). qRT-PCRs were performed in triplicate with 40 cycles. The relative gene expression was calculated using the 2^−△△CT^ method (Li et al., 2016), and GAPDH was used for mRNA template normalization.

### Histological Analysis

The mouse hearts were fixed in 4% paraformaldehyde and then embedded in paraffin. Paraffin-embedded sections were cut and stained with hematoxylin and eosin (H&E) to detect the morphology or 0.1% Sirius red to detect myocardial fibrosis. The oxidative fluorescent dye dihydroethidium (DHE; Invitrogen, Carlsbad, CA) was applied to 7 μm frozen sections of hearts at 40 μM for 30 min. Fluorescence intensity was measured and analyzed as previously described (Dai et al., 2018).

### Detection of MMP2 and MMP9 Activities

MMP2 and MMP9 activities were detected by using commercial kits (P1700, Applygen Technologies Inc., Beijing, China) according to the manufacturer’s instructions. Fifty micrograms of protein extracted from frozen tissues was electrophoresed in SDS-PAGE gels containing 1 mg/ml gelatin. Gels were washed in 2.5% Triton X-100 and incubated overnight in zymography developing buffer at 37°C. Then, the gels were stained with 0.5% Coomassie blue R250. Images were taken, and quantification was performed by Image Lab (Bio-Rad).

### ELISA

Follistatin protein levels in serum samples from patients, mouse heart tissues, and serum samples were measured using a human follistatin ELISA kit (EK0760, Boster China) following the manufacturer’s protocols.

### Determination of MnSOD

The MnSOD activity of mouse heart homogenates was measured using a superoxide dismutase (SOD) typed assay kit (hydroxylamine method) (Nanjing Jiancheng Bioengineering Institute, Nanjing, China) according to the manufacturer’s instructions.

### Determination of Malondialdehyde (MDA)

MDA content was determined by a lipid peroxidation MDA assay kit (Beyotime, S0131, China). After tissue homogenization with PBS, the products were centrifuged and the supernatants were blended with TBA detection solution and transferred to a 96-well plate to measure the absorbance at 532 nm, after which the MDA content was calculated according to the standard curve.

### Statistical Analysis

Data were analyzed using GraphPad Prism 6.0 software (GraphPad Software, San Diego, CA, United States) and expressed as the mean ± SEM. Student’s t tests were used for comparisons between two groups. For multiple groups, one-way analysis of variance was employed followed by Tukey’s *post hoc* tests. For all the analyses, a value of *p*<0.05 was considered significant.

## Results

### The Level of FST is Decreased in Diabetic Cardiomyopathy Patients and db/db Mice

**FIGURE 1:**
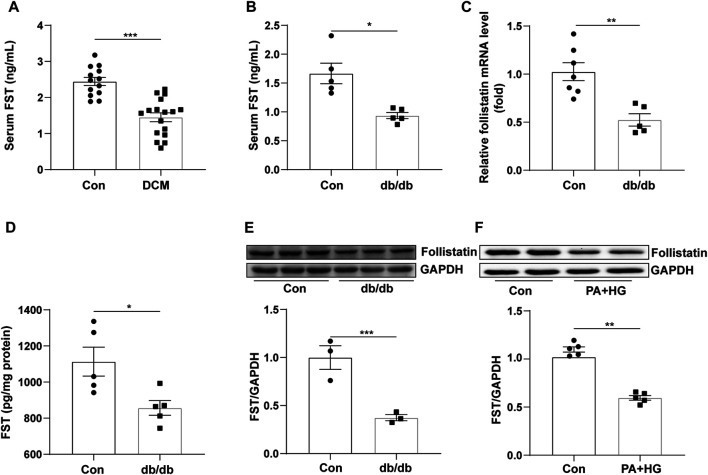
Relationship between FST and diabetic cardiomyopathy. Serum levels of FST in healthy controls and patients with diabetic cardiomyopathy [**(A)**, *n*≥13]. Levels of FST in serum [**(B)**, *n* = 5] and heart tissues of controls and db/db mice [**(C, D)**, *n*≥5]. Protein levels of FST in control and db/db mice [**(E)**, *n* = 3)]. Expression of FST in AC16 cells with or without HG + PA treatment [**(F)**, *n* = 5]. *p< 0.05; **p< 0.01; ***p<0.001.

To examine FST expression in diabetic cardiomyopathy, we assessed the level of FST in the serum of diabetic cardiomyopathy patients, and the FST level in db/db mouse serum and heart tissue was also assessed. We found that the level of FST in serum was significantly reduced by approximately 50% in diabetic cardiomyopathy patients and db/db mice compared with that in healthy controls (Figures 1A,B). PCR assays confirmed that the mRNA level of FST was decreased in the hearts of diabetic mice (Figure 1C). Western blot and ELISAs also showed that the cardiac expression of FST was significantly downregulated in db/db mice (Figures 1D,E). Additionally, to determine whether decreasing FST expression occurred *in vitro*, cultured AC16 cells were treated with palmitate plus glucose. Similarly, as compared with the control, the expression of FST in the treatment group was decreased (Figure 1F).

### Overexpression of FST Protects Against Cardiac Dysfunction in db/db Mice

**FIGURE 2:**
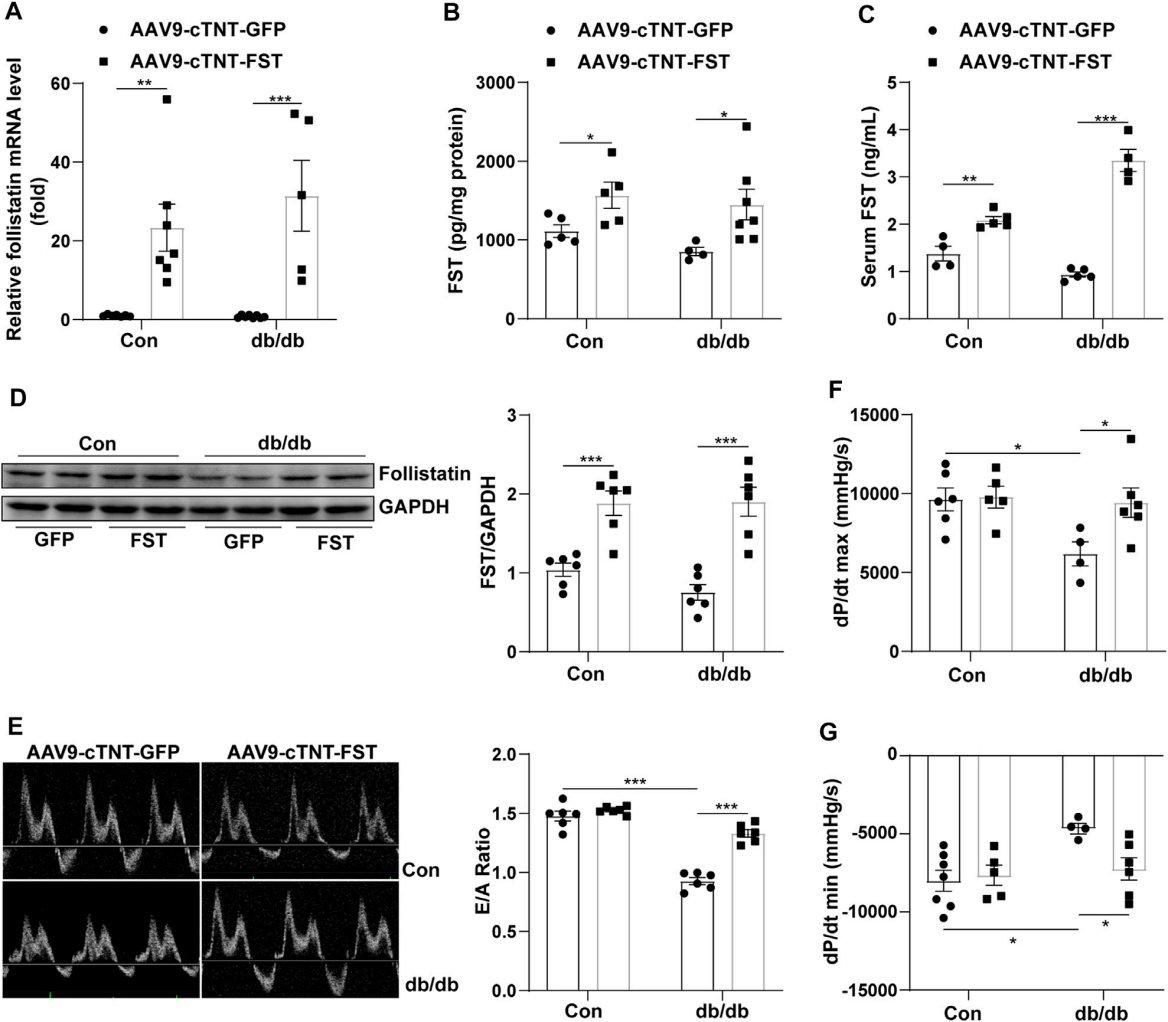
Overexpression of FST improves heart functions. Serum FST levels in controls and db/db mice [**(C)**, *n*≥4]. FST mRNA levels in mouse heart in each group [**(A)**, *n*≥5]. ELISA results of heart FST levels in each group [**(B)**, *n*≥4]. Western blot results of heart FST levels in each group [**(D)**, *n* = 6]. The ratio of E/A and echocardiograph images in each group [**(E)**, *n* = 6]. Hemodynamic analysis of db/db mice and controls [**(F,G)**, *n*≥4]. *p< 0.05; **p< 0.01; ***p<0.001.

To investigate the roles of FST in diabetic cardiomyopathy, we created db/db mice by using rAAV9 combined with the cTnT promoter delivery system. Cardiac functions were evaluated using transthoracic echocardiography and hemodynamic analysis. Compared with the AAV9-cTNT-GFP group, cardiac and serum FST levels were increased in AAV9-cTNT-FST–treated mice (Figures 2A–D). Although the mRNA level was increased by at least 20-fold, the protein level was increased by up to 1-fold following rAAV injection. In addition, compared with the control group (AAV9-cTNT-GFP), diabetes-induced cardiac dysfunction was reflected by a decreased E/A ratio, dP/dt maximum rate, and dP/dt minimum rate in the db/db mice injected with AAV9-cTNT-GFP (Figures 2E–G), but the EF value was not different between the two groups (Supplementary Table S1). However, FST overexpression reversed these changes in db/db mice. The E/A ratio, dP/dt maximum rate, and dP/dt minimum rate in the db/db mice with AAV9-cTNT-FST were elevated compared with those in the db/db with AAV9-cTNT-GFP group (Figures 2E–G). Moreover, increased body weight and blood glucose and decreased glucose tolerance power were observed in the db/db mice, but cardiac overexpression of FST had no effects on these metabolic characteristics (Supplementary Figure S1). Previous studies have convincingly demonstrated that FST promotes adipocyte differentiation, browning of white adipose tissue, and energy metabolism (Braga et al., 2014). Next, we assessed certain genes of lipid metabolism, including diacylglycerol O-acyltransferase 1 (DGAT1), CD36, acetyl-CoA carboxylase 2 (ACACB), acyl-CoA dehydrogenase (ACADS), carnitine palmitoyltransferase I (CPT1β), and peroxisomal acyl-coenzyme A oxidase 1 (ACOX1), and found that compared to the control AAV9-cTNT-GFP group, the synthesis of triglycerides, uptake and synthesis of fatty acid, and the β-oxidation of fatty acids increased in the hearts of db/db mice, which may induce lipid toxicity and oxidative stress, but overexpressing FST reduced the expression of these genes and then decreased the excessive accumulation of lipids (Supplementary Figures S2A–F).

### Overexpression of FST is Associated With Preserved Cardiac Hypertrophy

**FIGURE 3:**
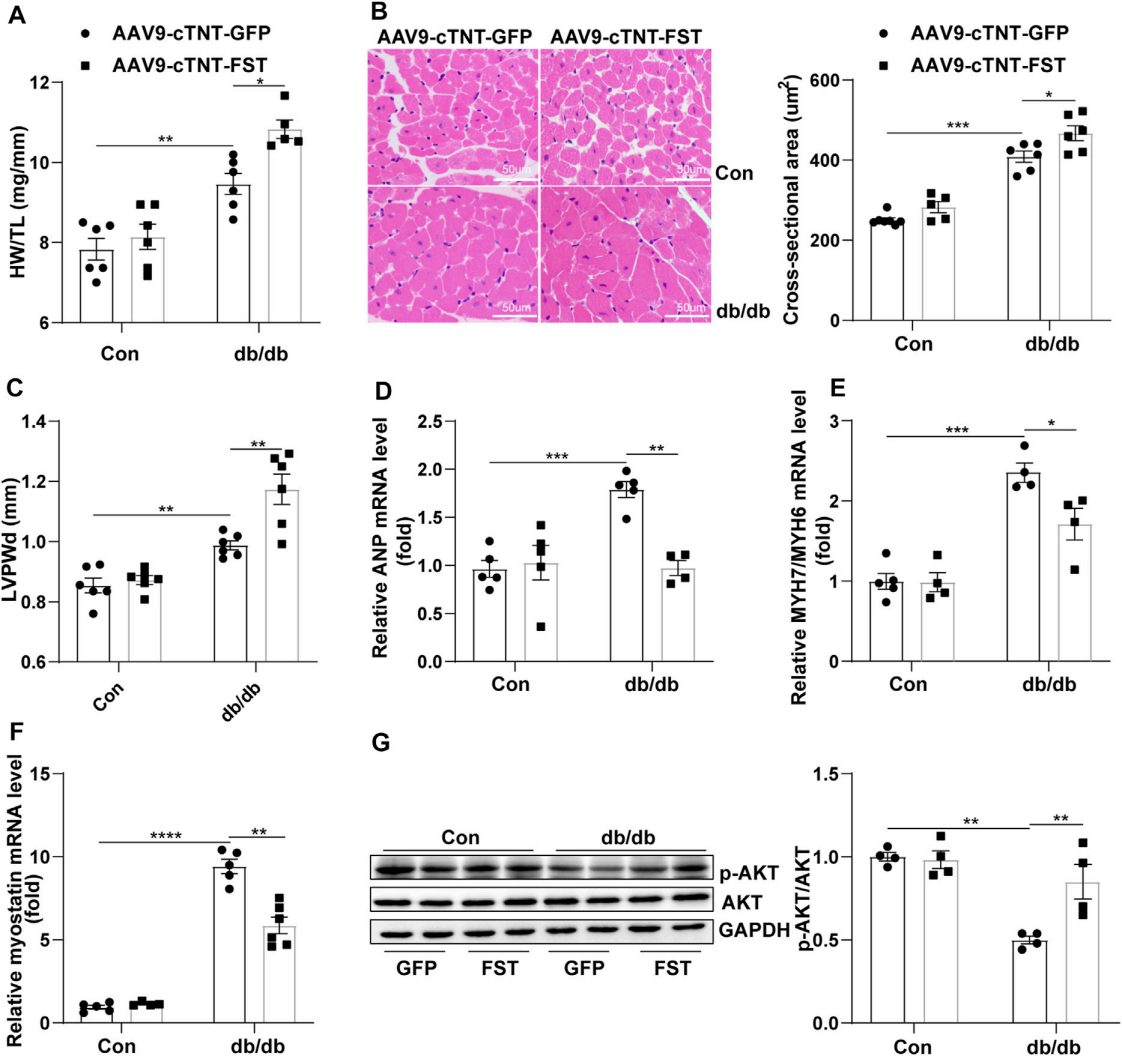
Overexpression of FST is associated with preserved cardiac hypertrophy. The ratio of heart weight/tibia length (HW/TL) of each group [**(A)**, *n* = 6]. Representative images of H&E staining and quantitative analysis (magnification: 400× [**(B)**, *n*≥5]). LVPWd was measured by echocardiography [**(C)**, *n*≥5]. Quantitative real-time PCR analysis of mRNA transcription of ANP, MYH7/MYH6 [**(D, E)**, *n*≥4], and myostatin [**(F)**, *n*≥4] in the myocardial tissues. Protein levels of *p*-AKT and AKT and quantitative analysis in heart tissues [**(G)**, *n* = 4]. *p< 0.05; **p< 0.01; ***p<0.001.

To elucidate how FST protected cardiac function, the heart weight, heart weight/tibia length (HW/TL), the cellular area, and some ultrasonic parameters were measured. The data show that the ratio of HW/TL and the cellular area were higher in db/db mice with AAV9-cTNT-GFP than those in db/db mice with the control-AAV9-cTNT-GFP, and overexpressing FST in db/db mice increased this ratio (Figures 3A,B). The echocardiography results were consistent with the above changes, such as LVPWd, LVPWs, and LV mass (Figure 3C, Supplementary Figure S2H;Supplementary Table S1). However, the RT-PCR results suggested that overexpressing FST in db/db mice decreased the mRNA levels of ANP and MYH7/MYH6 (Figures 3D,E). Previous studies have shown that FST increases muscle mass through the myostatin–AKT pathway (Gilson et al., 2009; Winbanks et al., 2012). Next, we determined the levels of myostatin, *p*-AKT, and AKT in each group. Compared with the control AAV9-cTNT-GFP group, db/db-AAV9-cTNT-GFP mice had higher myostatin and lower *p*-AKT/AKT levels, but overexpressing FST decreased myostatin and increased *p*-AKT/AKT in db/db mice (Figures 3F,G). These data indicated that overexpressing FST might promote cardiac hypertrophy in a compensatory manner by downregulating myostatin and upregulating *p*-AKT.

### Overexpression of FST Reduces Cardiac Fibrosis and Enhances MMP9 Activity in db/db Mice

**FIGURE 4:**
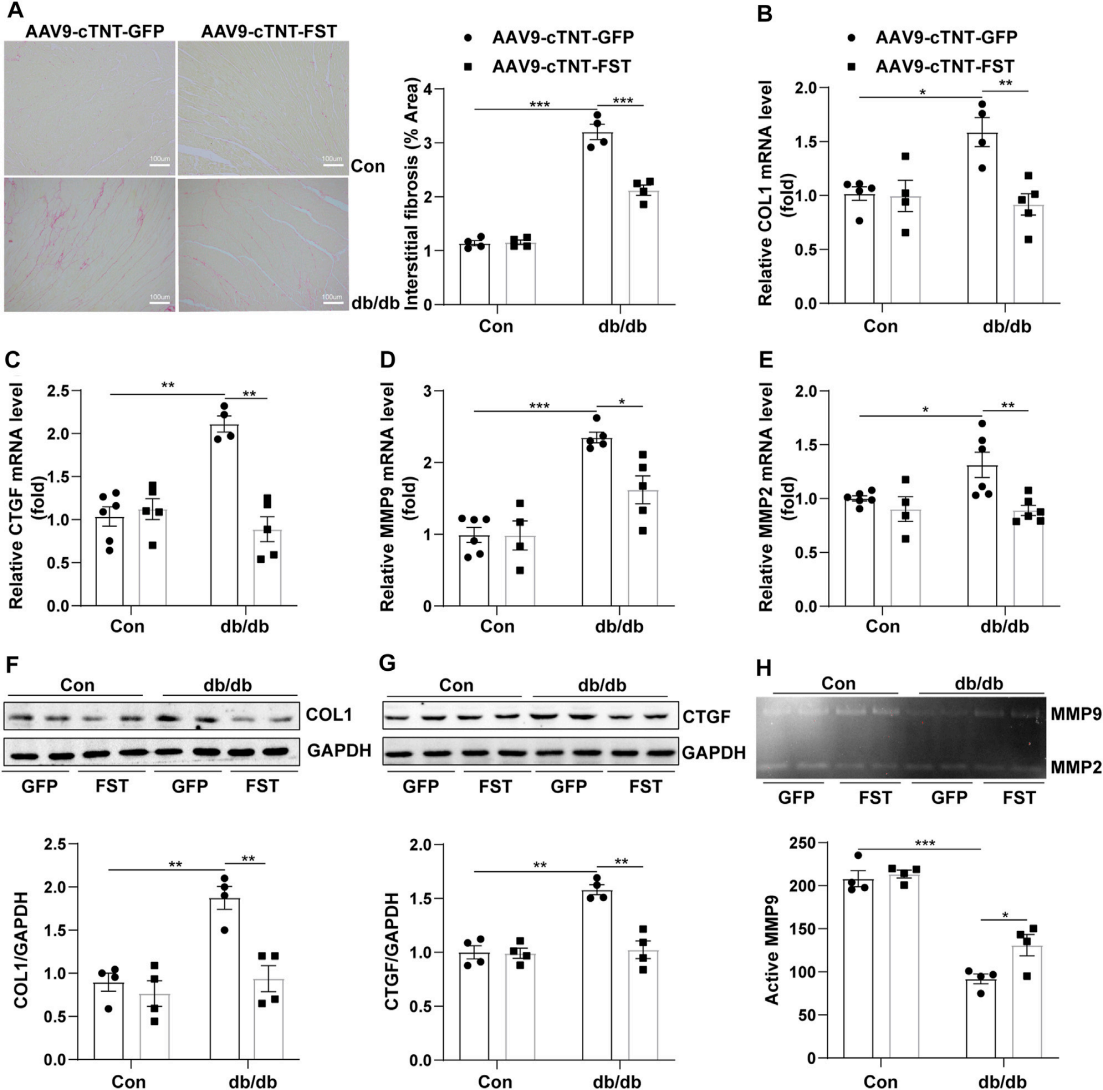
Overexpression of FST abrogates cardiac fibrosis. Representative images of Sirius red staining and quantitative analysis of myocardial interstitial collagen (magnification: 200×[**(A)**, *n* = 4]). Quantitative real-time PCR analysis of mRNA transcription of fibrosis markers COL1 and CTGF [**(B, C)**, *n*≥4]. Western blotting and quantitative analysis of COL1 and CTGF [**(F, G)**, *n* = 4]. Quantitative real-time PCR analysis of mRNA transcription of MMP9 and MMP2 [**(D, E)**, *n*≥4] and activity measured by zymography [**(H)**, *n* = 4] in each group. *p< 0.05; **p< 0.01; ***p<0.001.

To determine whether FST influenced fibrosis in diabetic hearts, the myocardial collagen content was examined by Sirius red staining. Histological examination revealed that the fibrotic area of the myocardial interstitium in the db/db-AAV9-cTNT-GFP group increased significantly compared to that in the control-AAV9-cTNT-GFP group. This increase was significantly inhibited in db/db-AAV9-cTNT-FST hearts compared to that of control-AAV9-cTNT-GFP hearts (Figure 4A). Next, we examined the expression of fibrosis-related genes by Western blot and RT-PCR assays. The corresponding detection results showed that the expression of fibrotic markers, such as collagen-I and connective tissue growth factor (CTGF), was dramatically increased in diabetic hearts but was retarded by overexpressing FST (Figures 4B,C,F,G). Because matrix metalloproteinase-2 (MMP2) and matrix metalloproteinase-9 (MMP9) are associated with fibrosis in diabetic cardiomyopathy (Biernacka et al., 2015), we examined the expression and activity of MMP2 and MMP9. The RT-PCR results showed that compared with the control-AAV9-cTNT-GFP group, the relative mRNA levels of MMP2 and MM9 increased in the db/db-AAV9-cTNT-GFP group, and overexpressing FST decreased this tendency (Figures 4D,E). However, gelatin zymography showed that compared with the control-AAV9-cTNT-GFP, the activity of MMP9 was reduced in the db/db-AAV9-cTNT-GFP group, and overexpressing FST increased MMP activity. The activity of MMP2 was not different between the groups (Figure 4H, Supplementary Figure S2G).

These results suggested that FST decreased cardiac fibrosis in db/db mice and enhanced MMP9 activity.

### FST Reduces Cardiac Fibrosis by inhibiting the Activation of the TGF-β–Smad Pathway

**FIGURE 5:**
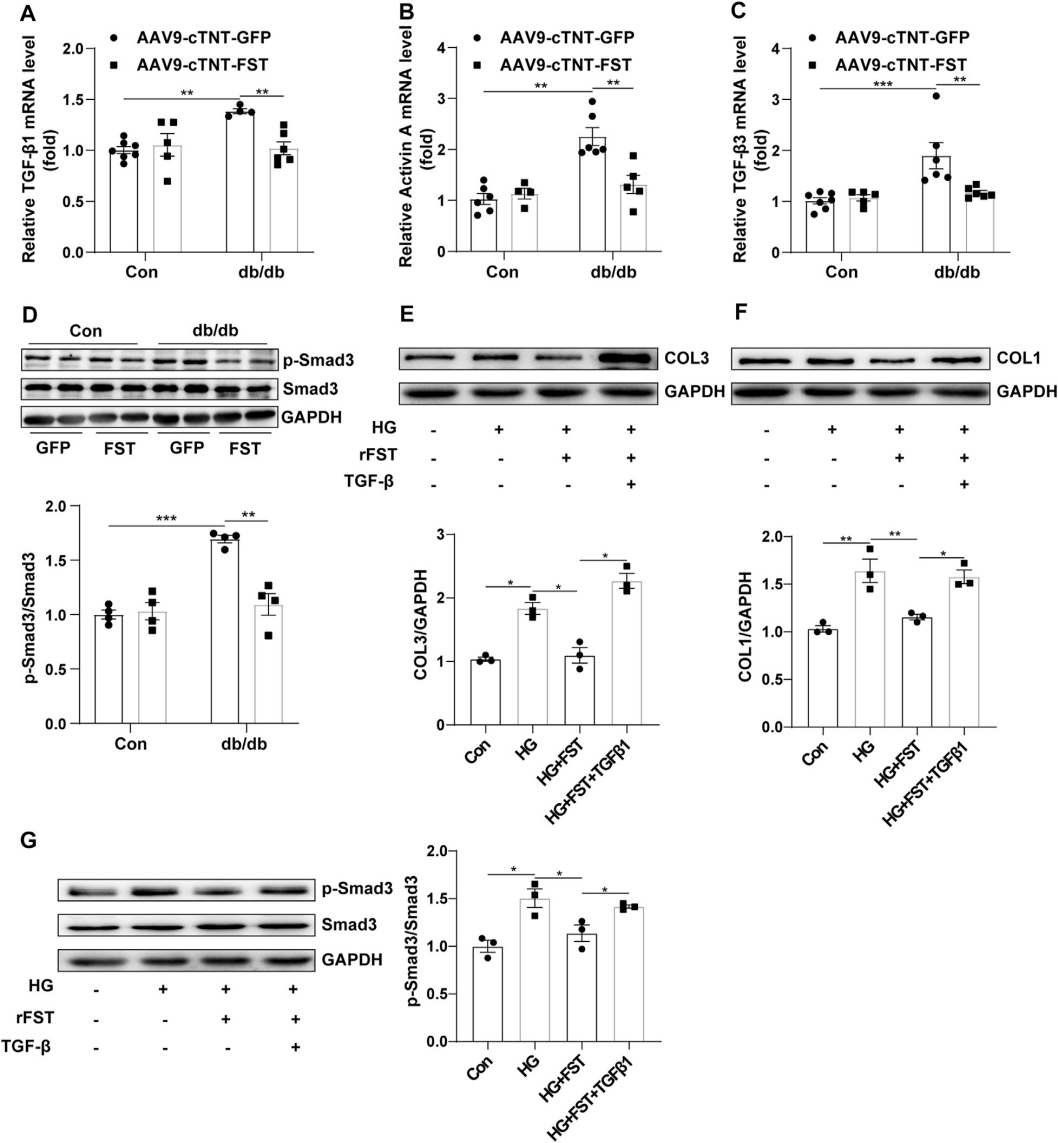
Overexpression of FST reduces cardiac fibrosis by inhibiting the activation of the TGF-β–Smad3 pathway *in vivo* and *in vitro*. Quantitative real-time PCR analysis of TGF-β1, activin A, and TGF-β3 mRNA transcription [**(A–C)**, *n*≥4] and *p*-Smad3 and Smad3 protein expression in each group [**(D)**, *n* = 4]. *In vitro*, NIH3T3 cells were treated with or without high glucose (30 mM), recombinant FST protein (10 ng/ml), and TGF-β1 (20 μM). Protein expression of COL1, COL3, *p*-Smad3, and Smad3 with different treatments [**(E–G)**, *n* = 3]. *p< 0.05; **p< 0.01; ***p<0.001.

Previous studies have shown that the TGF-β superfamily is associated with fibrosis (Yuan Ma et al., 2017). To determine whether FST attenuated myocardial fibrosis by downregulating the TGF-β–Smad pathway, we assessed some members of the TGF-β superfamily. The results showed that compared with the control-AAV9-cTNT-GFP group, the mRNA levels of TGF-β1, activin A, and TGF-β3 increased in db/db-AAV9-cTNT-GFP mice; however, overexpressing FST in db/db mice significantly decreased these levels of the members (Figures 5A–C). Western blot analysis indicated that compared with the control-AAV9-cTNT-GFP, the phosphorylation of Smad3 and total Smad3 was increased in the db/db-AAV9-cTNT-GFP group, but overexpressing FST significantly inhibited the phosphorylation of Smad3 and decreased the expression of total Smad3 (Figure 5D). Next, to verify the above result, an *in vitro* experiment was performed, and the results suggested that high glucose treatment increased the level of COL1 and COL3, and a low dose of recombinant FST (10 ng/ml, 100 ng/ml) reduced the expression of both, but a high dose of FST (500 ng/ml) had no effect (Figure S2I). In an *in vitro* experiment, FST not only decreased the COL1 and COL3 induced by high glucose but also reduced *p*-SMAD3/SMAD3. However, this protective effect disappeared when TGF-β was added to the cells (Figures 5E–G).

Therefore, the above results indicated that FST might attenuate cardiac fibrosis in diabetic cardiomyopathy by inhibiting the TGF-β–Smad3 pathway.

### Overexpression of FST Decreases Cardiac Oxidative Stress in db/db Mice

**FIGURE 6:**
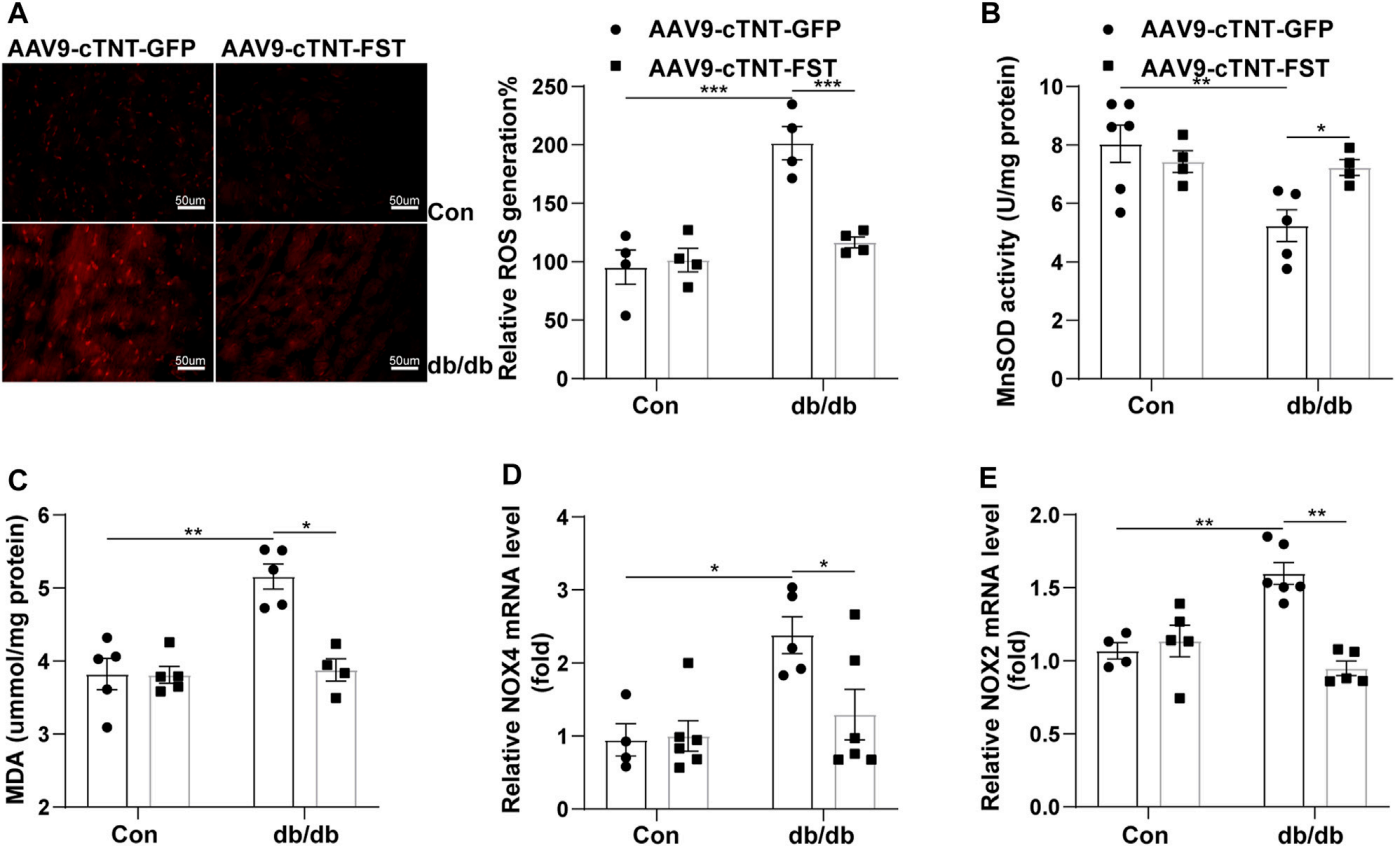
Overexpression of FST decreases cardiac oxidative stress in db/db mice. Representative images of DHE staining and quantitative analysis of myocardial ROS levels in each group (magnification: 400×[**(A)**, *n* = 4]). The activity of MnSOD, the content of MDA, and the quantitative real-time PCR analysis of mRNA transcription of NOX4 and NOX2 in heart tissues (B–E, *n*≥4).*p< 0.05; **p< 0.01; ***p<0.001.

Oxidative stress plays an important role in diabetic cardiomyopathy (Natali et al., 2017; Lytrivi et al., 2018). Dihydroethidium (DHE) staining showed that the level of reactive oxygen species (ROS) significantly increased in the hearts of db/db- AAV9-cTNT-GFP mice compared with the control-AAV9-cTNT-GFP group. The changes in malondialdehyde (MDA) content and the expression of NADPH oxidase 4 (NOX4) and NADPH oxidase 2 (NOX2) in the heart were similar to those of ROS. However, overexpression of FST in db/db mice reduced the levels of ROS, MDA, NOX4, and NOX2 (Figures 6A,C–E). Next, we assessed the activity of MnSOD. In db/db mice, the activity of MnSOD was reduced, and overexpressing FST increased its activity (Figure 6B). The above findings showed that FST reduced cardiac oxidative stress in db/db mice.

## Discussion

**FIGURE 7:**
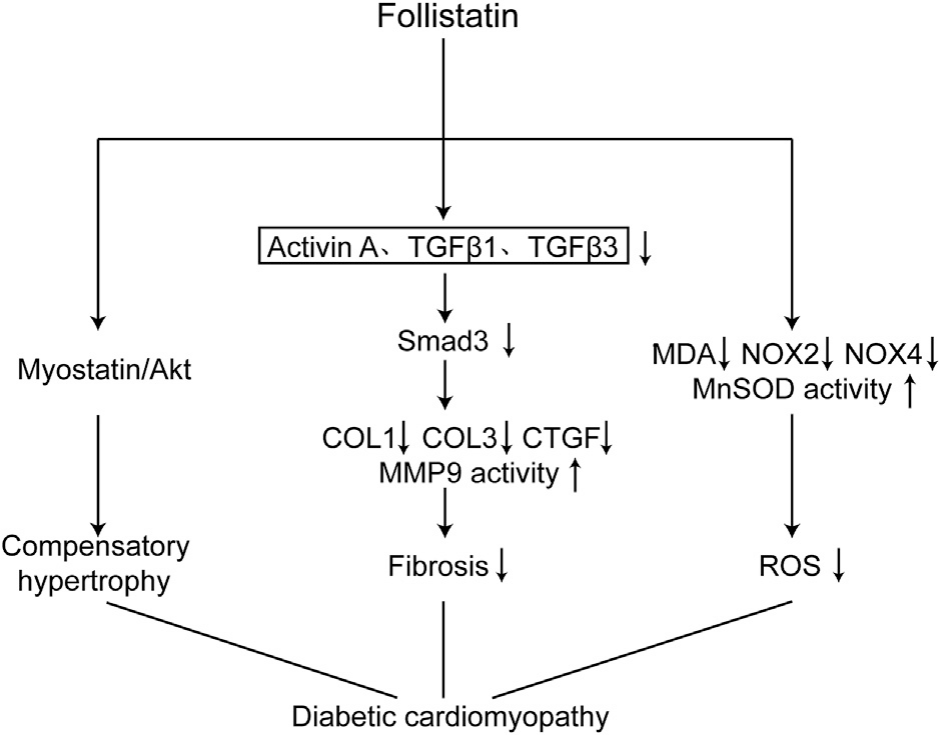
Schematic diagram of the mechanism of FST in improving diabetic cardiomyopathy. Overexpression of FST in the heart attenuate diabetic cardiomyopathy through the following ways: causing compensatory hypertrophy by the Myostatin/Akt pathway, reducing fibrosis through the TGF-β/Smad3 pathway, and decreasing oxidative stress by increasing the scavenging capacity of reactive oxygen species and reducing the production of reactive oxygen species.

Our study has demonstrated that FST is an important molecule involved in the development of diabetic cardiomyopathy. Overexpression of FST reversed cardiac dysfunction, reduced fibrosis, and inhibited oxidative stress in the diabetic heart(Figure 7).

Previous studies have shown that circulating FST is higher in patients with T2D than in glucose-tolerant individuals or obese individuals and that activin A levels are similar between these groups (Perakakis et al., 2019; Sylow et al., 2020). However, our results indicated that the level of serum FST was lower in patients with diabetic cardiomyopathy than in healthy individuals, and the *in vitro* experimental results also verified this finding. FST could bind and block the function of activin A; thus, the increased ratio of activin A/FST may suggest increased signaling through activin A–activated mechanisms in diabetic cardiomyopathy. In addition, although FST correlates with insulin resistance in patients with T2D, the effect of insulin on reducing circulating FST is preserved in obese individuals and patients with T2D. Moreover, with an increasing insulin exposure time, FST mRNA levels in human hepatocytes decrease gradually (Sylow et al., 2020). Therefore, the decline in the level of FST in diabetic cardiomyopathy might be explained by prolonged exposure to insulin. However, further experiments are needed to address this hypothesis.

We also found that cardiac-specific overexpression of FST *via* tail intravenous injection improved the cardiac function of db/db mice but had no effect on weight, plasma glucose, or glucose tolerance. The technique of using rAAV combined with the cTnT promoter delivery system to realize overexpression of specific genes has been used broadly. In our study, the cardiac function of db/db mice deteriorated, specifically reflected by a decreased E/A ratio and values of dP/dt max and dP/dt min, which were consistent with previous studies (Fein, 1990; Huynh et al., 2014; Li et al., 2019). Moreover, overexpression of FST reversed these reduced changes, which shows a beneficial effect on improving the dysfunction of diabetic cardiomyopathy. Moreover, although the results indicated that FST improves cardiac function, there was no effect on metabolic parameters, such as weight, plasma glucose, fasting glucose, and glucose tolerance. We speculate that overexpression of FST in the heart might have no effect on general metabolism.

In addition, our results also revealed that FST overexpression increases cardiac hypertrophy in a compensatory manner. In our experiment, db/db mice developed cardiac hypertrophy, including an increased ratio of HW/TL, average cardiac myocyte cross-sectional area, and other ultrasonic parameters, similar to changes at the molecular level, which was consistent with previous studies (Huynh et al., 2014; Dai et al., 2018; Jia et al., 2018). However, FST overexpression increased hypertrophy but did not change the levels of ANP or MYH7/MYH6. Previous research has reported that FST is associated with skeletal muscle hypertrophy with increased activation of AKT phosphorylation levels dependent on or independent of myostatin (Gilson et al., 2009; Winbanks et al., 2012). Furthermore, physiological activation of AKT protects against pathological hypertrophy (Moc et al., 2015), and chronic AKT blockade aggravates pathological hypertrophy and inhibits physiological hypertrophy (Buss et al., 2012). By detecting myostatin and *p*-AKT in each group, we found decreased phosphorylation levels of AKT in db/db mice and that FST increased the changes. Therefore, we speculate that FST protects against the cardiac function of diabetic cardiomyopathy by increasing cardiac hypertrophy in a compensatory manner, similar to physiological hypertrophy. More detailed experiments are needed to confirm this.

Moreover, we also found that fibrosis was involved in the development of diabetic cardiomyopathy, including collagen accumulation in the myocardial interstitium; increased expression of COL1, COL3, or CTGF at the transcriptional and protein levels; and reduced activity of MMP9, which were consistent with previous studies (Fein, 1990; Shimizu, 1993; Mizushige et al., 2000: Westermann et al., 2007; Linthout et al., 2008; Braga et al., 2014; Huynh et al., 2014). In addition, the TGF-β–Smad3 pathway plays an important role in the pathogenesis of diabetic cardiomyopathy (Braga et al., 2014; Yuan Ma et al., 2017). *In vivo*, we found increased levels of TGF-β1, activin A, and TGF-β3, which are members of the TGF-β superfamily, and decreased phosphorylation of AKT. However, overexpressing FST reduced the levels of the above increased molecules, including decreased collagen accumulation and altered expression of a number of fibrosis markers. The anti-fibrotic effects of FST have been studied in other organs (Ohnishi, 2003; Aoki et al., 2005; Patella et al., 2006; Pons et al., 2018). Although the study has reported that FST does not bind to TGF-β1 or 2 (Nogai et al., 2008), the anti-fibrotic activity of FST seems to be attributable to the inhibition of activin A produced by cells in response to TGF-β1 (Hedger and de Kretser, 2013). Our *in vitro* studies also confirmed that FST decreased the levels of COL1, COL3, and *p*-Smad3 after treatment with high glucose. Interestingly, treatment with high concentrations of FST protein *in vitro* increased the expression of COL and COL3, possibly due to excessive inhibition of the TGF-β superfamily, but more experiments are needed for verification. Additionally, we found that overexpressing FST reduced oxidative stress in db/db mice; downregulated the levels of NOX4, NOX2, and MDA; and increased the activity of MnSOD. Other studies have also shown that FST protects against ROS production in several settings, both *in vitro* and *in vivo* (Zhang et al., 2018), dependent on or independent of activin A in other organs excluding diabetic hearts. Although the effect of FST on reducing oxidative stress has been found in diabetic cardiomyopathy, more detailed mechanisms need to be found to better verify the antioxidant activity.

However, our research did not study the specific binding sites of FST, such as activin A, TGF-β1, or TGF-β3. In addition, we did not conduct in-depth research on how FST influences lipid metabolism, and the detailed mechanisms of antioxidant activity have not been researched.

Despite some limitations, our data support a novel role of FST in improving diabetic cardiomyopathy by reducing fibrosis and oxidative stress. Our results suggested that FST may be a promising therapeutic target for treating diabetic cardiomyopathy.

## Conclusion

Our findings suggested that FST plays an important role in improving diabetic cardiomyopathy by its anti-fibrosis effects through the TGF-β–Smad3 pathway. According to our study, FST is expected to become a new treatment for diabetic cardiomyopathy.

## Data Availability

The original contributions presented in the study are included in the article/Supplementary Material; further inquiries can be directed to the corresponding author.
