# Supplementary material for: Follistatin Attenuates Myocardial Fibrosis in Diabetic Cardiomyopathy via the TGF-β–Smad3 Pathway
Source: Front Pharmacol. 2021 Jul 27;12:683335. doi: 10.3389/fphar.2021.683335 (PMC8353454; doi:10.3389/fphar.2021.683335)
Supplement: Supplementary file 1 [file table1.docx]

**TableS1** **Echocardiographic data at 8 weeks after injection of Adeno-Associated Viral** **Vector**

|  | Con | | db/db | |
| --- | --- | --- | --- | --- |
|  | AAV9-cTNT-GFP | AAV9-cTNT-FST | AAV9-cTNT-GFP | AAV9-cTNT-FST |
| HR (bpm) | 540±9 | 529±17 | 521±5 | 508±13 |
| EF (%) | 78.17±2.23 | 77.35±3.01 | 75.29±0.98 | 79.37±1.61 |
| FS (%) | 46.43±2.12 | 44.89±3.74 | 43.53±0.91 | 47.55±1.55 |
| LV Mass (mg) | 124.87±6.89 | 125.81±3.13 | 149.52±8.74***** | 174.93±10.43& |
| CO (mL/min) | 24.26±2.53 | 22.93±1.49 | 25.81±1.89 | 26.52±0.67 |
| LVAW; d (mm) | 0.84±0.04 | 0.91±0.02 | 1.07±0.02***** | 1.25±0.06& |
| LVAW; s (mm) | 1.32±0.05 | 1.40±0.05 | 1.66±0.05***** | 1.70±0.07 |
| LVID; d (mm) | 3.82±0.17 | 3.81±0.07 | 3.86±0.16 | 3.83±0.14 |
| LVID; s (mm) | 2.16±0.11 | 2.17±0.07 | 2.10±0.12 | 1.81±0.2 |

*P < 0.05versus Con -AAV9-cTNT-GFP; & P < 0.05versus db/db-AAV9-cTNT-GFP
