# Supplementary figures and images for: Follistatin Attenuates Myocardial Fibrosis in Diabetic Cardiomyopathy via the TGF-β–Smad3 Pathway
Source: Front Pharmacol. 2021 Jul 27;12:683335. doi: 10.3389/fphar.2021.683335 (PMC8353454; doi:10.3389/fphar.2021.683335)

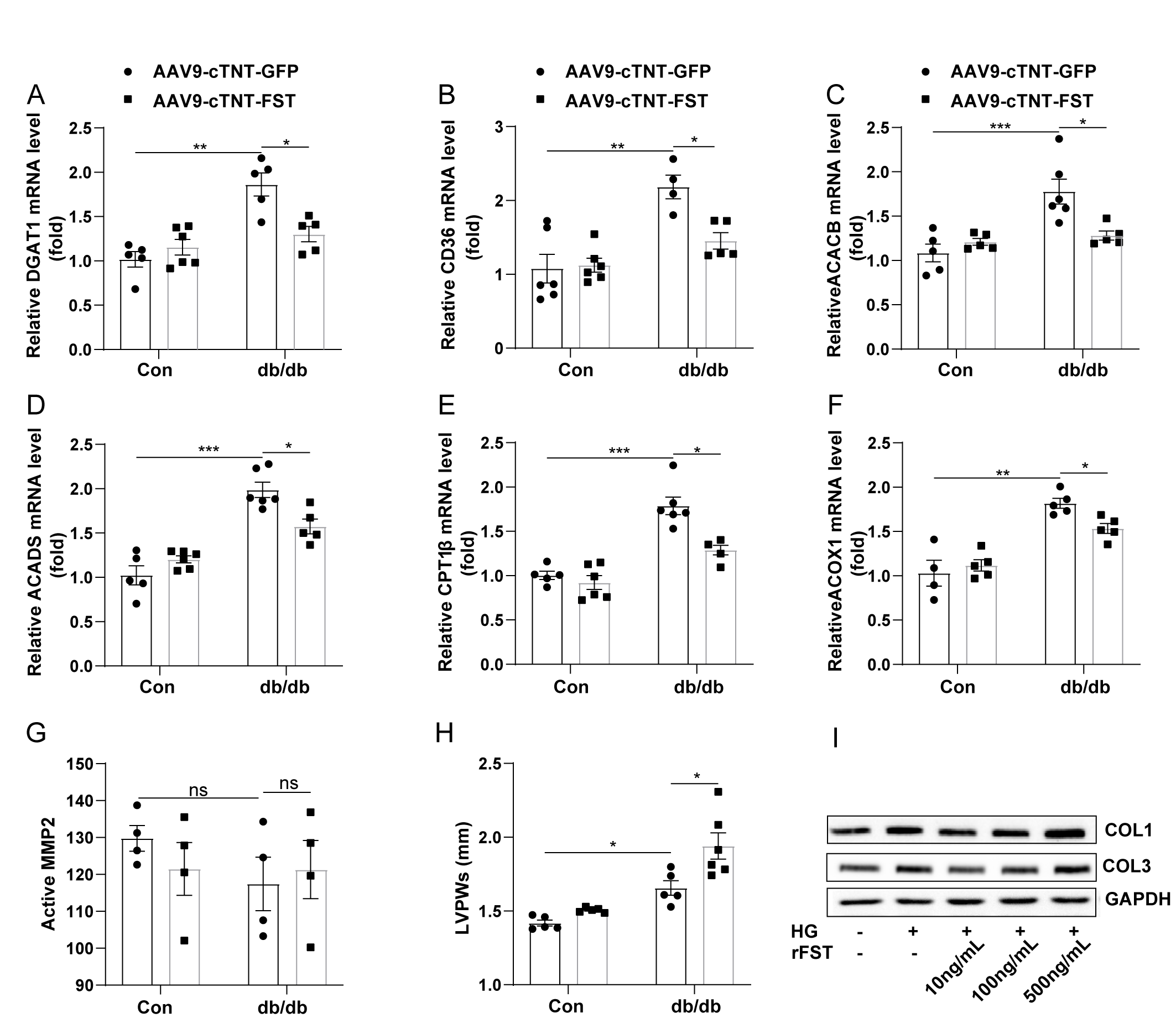

Supplement: Supplementary file 2 [file image2.tif]

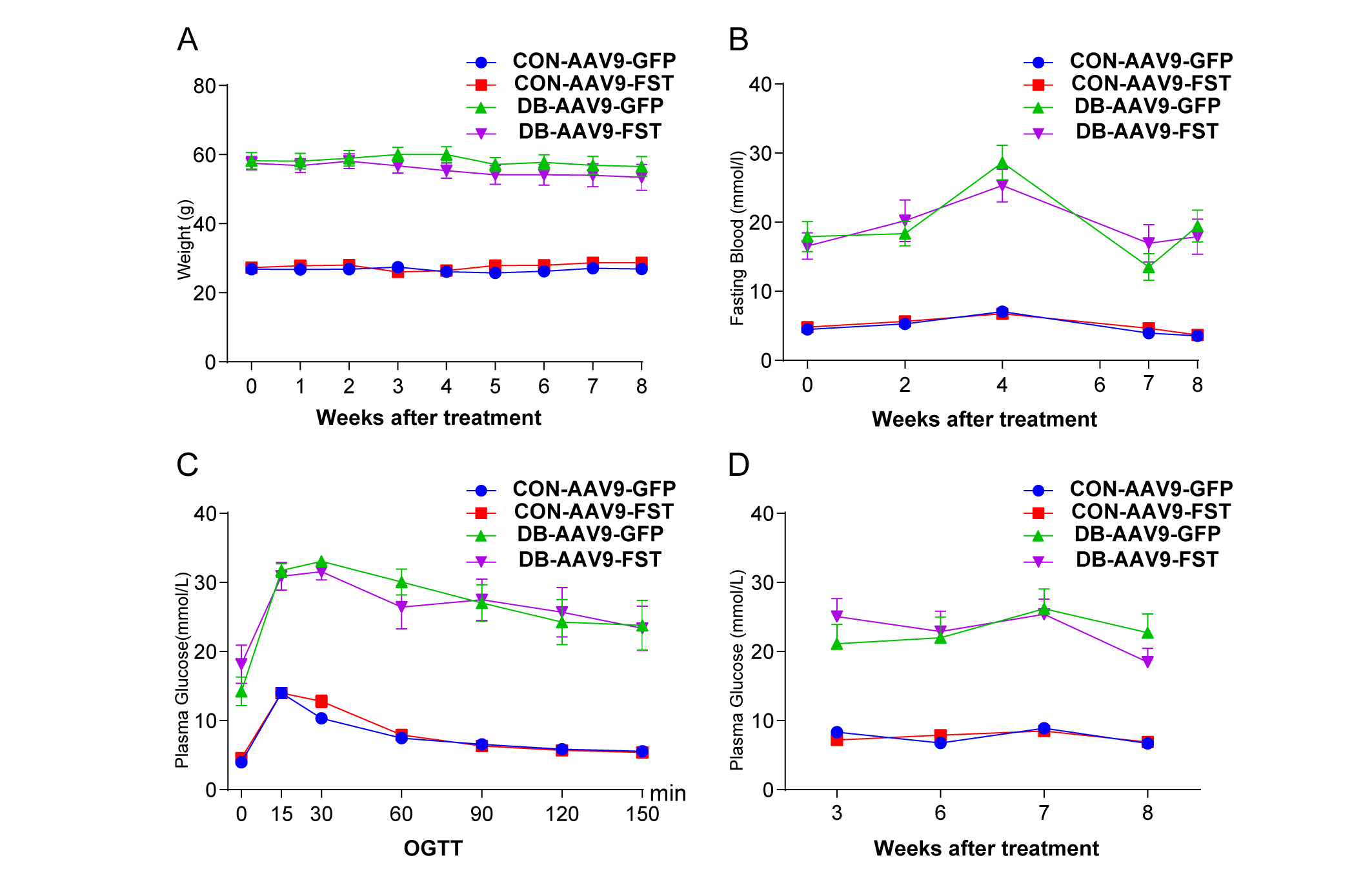

Supplement: Supplementary file 3 [file image1.tif]
