# Supplementary material for: Follistatin Attenuates Myocardial Fibrosis in Diabetic Cardiomyopathy via the TGF-β–Smad3 Pathway
Source: Front Pharmacol. 2021 Jul 27;12:683335. doi: 10.3389/fphar.2021.683335 (PMC8353454; doi:10.3389/fphar.2021.683335)
Supplement: Supplementary file 4 [file table2.docx]

**Table S2 Sequences and sources of PCR primers**

| **Primer name** | **Sequence (5'-3')** | **Company** |
| --- | --- | --- |
| mFST-F | TCAAAGCAAAGTCCTGTGAAGA | TsingKe |
| mFST -R | TGTCAGGACACAGCTCATCG | TsingKe |
| mANP-F | AGGCAGTCGATTCTGCTTGA | TsingKe |
| mANP-R | CGTGATAGATGAAGGCAGGAAG | TsingKe |
| mα-MHC-F | CTGTCCAAGTTCCGCAAGGT | TsingKe |
| mα-MHC-R | TCGTGCATCTTCTTGGCACC | TsingKe |
| mβ-MHC-F | AGCCTCAGCAGAGGAGTACA | TsingKe |
| mβ-MHC-R | GGCTGAGCCTTGGATTCTCA | TsingKe |
| mGAPDH-F | GACCTCATGGCCTACATGGC | TsingKe |
| mGAPDH-R | ATTATGGGGGTCTGGGATGGA | TsingKe |
| mCol1-F | GCTCCTCTTAGGGGCCACT | TsingKe |
| mCol1-R | CCACGTCTCACCATTGGGG | TsingKe |
| mCTGF-F | ACTATGATGCGAGCCAACTGC | TsingKe |
| mCTGF-R | TGTCCGGATGCACTTTTTGC | TsingKe |
| mMMP2-F | CAAGTTCCCCGGCGATGTC | TsingKe |
| mMMP2-R | TTCTGGTCAAGGTCACCTGTC | TsingKe |
| mMMP9-F | CAGCCGACTTTTGTGGTCTTC | TsingKe |
| mMMP9-R | GGTACAAGTATGCCTCTGCCA | TsingKe |
| mActivinA-F | TGGTGCCAGTCTAGTGCTTC | TsingKe |
| mActivinA -R | CCGTCACTCCCATCTTTCTT | TsingKe |
| mMyostatin-F | AGTGGATCTAAATGAGGGCAGT | TsingKe |
| mMyostatin -R | GTTTCCAGGCGCAGCTTAC | TsingKe |
| mTGFβ1-F | CTCCCGTGCTTCTAGTGC | TsingKe |
| mTGFβ1-R | GCCTTAGTTTGGACAGGATCTG | TsingKe |
| mTGFβ3-F | CCTGGCCCTGCTGAACTTG | TsingKe |
| mTGFβ3-R | TTGATGTGGCCGAAGTCCAA | TsingKe |
| mACOX1- F | TAACTTCCTCACTCGAAGCCA | TsingKe |
| mACOX1- R | AGTTCCATGACCCATCTCTGTC | TsingKe |
| mACADs-F | TGGCGACGGTTACACACTG | TsingKe |
| mACADs-R | GTAGGCCAGGTAATCCAAGCC | TsingKe |
| mCPT1β-F | GCACACCAGGCAGTAGCTTT | TsingKe |
| mCPT1β-R | CAGGAGTTGATTCCAGACAGGTA | TsingKe |
| mCD36-F | ATGGGCTGTGATCGGAACTG | TsingKe |
| mCD36-R | GTCTTCCCAATAAGCATGTCTCC | TsingKe |
| mACACβ-F | CCTTTGGCAACAAGCAAGGTA | TsingKe |
| mACACβ-R | AGTCGTACACATAGGTGGTCC | TsingKe |
| mDGAT1 -F | TCCGTCCAGGGTGGTAGTG | TsingKe |
| mDGAT1-R | TGAACAAAGAATCTTGCAGACGA | TsingKe |
