# Supplementary material for: Follistatin Attenuates Myocardial Fibrosis in Diabetic Cardiomyopathy via the TGF-β–Smad3 Pathway
Source: Front Pharmacol. 2021 Jul 27;12:683335. doi: 10.3389/fphar.2021.683335 (PMC8353454; doi:10.3389/fphar.2021.683335)
Supplement: Supplementary file 5 [file datasheet1.docx]

# Supplemental figure legends

**Figure S1. Effect of FST on glycemic parameters.** Time courses of weight, random and fasting blood glucose levels. The measurements were made periodically from control and db/db mice (A-C, n=8). OGTTs (2g/kg glucose) were performed in each group (n=8). *p < 0.05; **p <0.01; ***p <0.001

**Figure S2. Effect of FST on lipid metabolism 、LVPWs、activity of MMP2 in vivo and levels of Col1、Col3 with recombinant FST protein treatment.** Quantitative realtime PCR analysis of mRNA transcription for lipid metabolism gene in each group, including synthesis of triglycerides（A, n≥5）、uptake and synthesis of fatty acid (B-C, n≥5) and β oxidation of fatty acids（D-F, n≥5.）Activity of MMP2 was measured by zymography (G, n=4). LVPWs was measured by echocardiography (H n≥5). The protein level of Col1、Col3 in a concentration range of recombinant follistatin protein (10-500ng/ml) (I) *p < 0.05; **p < 0.01; ***p <0.001

**TableS1** **Echocardiographic data at 8 weeks after injection of Adeno-Associated Viral** **Vector** *P < 0.05versus Con -AAV9-cTNT-GFP; & P < 0.05versus db/db-AAV9-cTNT-GFP

**TableS2 Sequences and sources of PCR primers**
